# Thriving in the First 1000 Days: Lessons from Positive Deviance Among Young Families

**DOI:** 10.3390/ijerph22101600

**Published:** 2025-10-21

**Authors:** Andrew P. Hills, Sisitha Jayasinghe, Kylie Mulcahy, Nuala M. Byrne

**Affiliations:** 1 UTAS Health, School of Health Sciences, University of Tasmania, Launceston, TAS 7250, Australia; nuala.byrne@utas.edu.au; 2 Independent Researcher, Newcastle NE1, UK; sisitha.jay@gmail.com; 3 Burnie Works, Burnie, TAS 7320, Australia; kmulcahy@burnieworks.com.au

**Keywords:** first 1000 days, positive deviance, community-led change, child health

## Abstract

The first 1000 days (F1D), from conception to a child’s second birthday, constitute a critical window for shaping long-term health, development, and wellbeing. While conventional approaches often rely on external interventions to support young families, the Positive Deviance (PD) framework offers a compelling alternative: identifying and amplifying successful behaviours already present within communities facing similar constraints. This paper explores how PD can be harnessed to foster sustainable, community-led change during the F1D. By uncovering local success stories, promoting participatory engagement, and strengthening caregiver self-efficacy, PD enables communities to co-create solutions that are culturally relevant and contextually grounded. However, effective application of PD requires careful attention to structural inequities, ethical storytelling, and rigorous methodological standards to avoid inadvertently shifting responsibility onto individuals. When implemented thoughtfully, PD reveals “what works” in resource-limited settings, empowering communities to build child-inclusive environments rooted in local expertise and resilient practices.

## 1. Introduction

The first 1000 days (F1D), from conception to a child’s second birthday, represent a critical developmental phase during which foundational aspects of health, nutrition, and social wellbeing are established. Ensuring optimal conditions during this period is essential to lifelong outcomes. Yet, conventional approaches to early childhood support often rely on external interventions, overlooking the latent potential within communities themselves.

Positive Deviance (PD), a concept rooted in identifying successful behaviours and practices among individuals who thrive despite facing similar constraints, has demonstrated efficacy across diverse health domains. These include infection prevention and control, medication safety, surgical outcomes, mental health stigma reduction, reproductive health, and chronic disease management [1]. PD may also offer an innovative, locally driven pathway to improve child health and development outcomes during the F1D.

By recognizing and amplifying uncommon yet effective behaviours, communities can foster inclusive, supportive environments for young families. PD is particularly promising in contexts marked by deep health inequities, where conventional health promotion efforts have yielded limited gains. From the perspective of community-based health activism, PD represents a paradigm shift, from deficit-focused models that emphasize what is lacking, to asset-based frameworks grounded in lived success. It reframes the outlier as a guide: evidence that even within constrained environments, sustainable pathways to better health and equity are possible [2,3]. To this end, PD is distinct in that it focuses on identifying uncommon yet successful behaviours within a community to address a specific problem. This contrasts with community-based participatory research (CBPR), which emphasizes equitable, collaborative research approaches to tackle community-identified issues, and with asset-based community development (ABCD), which centres on identifying and mobilizing all available community assets for problem-solving [4,5]. Conversely, once set in motion, PD can be understood as a catalyst for ‘social innovation’, as it identifies and amplifies solutions that already exist within the community, allowing them to spread organically [6]. Moreover, once these ‘outliers’ are recognised, their behaviours often diffuse naturally through peer influence, storytelling, and shared practice, driving wider transformation without external imposition, as theorised in the concept of ‘community diffusion of behaviour’ [7]. In either case, the merit and transformative potential of PD are indisputable.

Moreover, in a global landscape where many health systems struggle with quality, especially in resource-limited settings, PD offers a compelling alternative. Its minimal reliance on external resources and emphasis on existing community strengths make it a powerful tool for scalable, contextually relevant change [1].

## 2. The F1D and Positive Deviance

The F1D is widely recognized as a critical “window of opportunity” during which the foundations for lifelong health, development, and wellbeing are established. The quality of connections, nutrition, nurturing care, and movement experiences a child receives during this period significantly influences future outcomes in education, health, and social participation. A central global and local challenge remains—how to build child-inclusive societies where every child has the opportunity to thrive, beginning with the F1D [8].

In today’s hyperconnected world, individuals frequently seek health-related information from external sources, particularly online platforms. When it comes to decisions about child development and wellbeing, communities often look outward for guidance, validation, or solutions to local challenges. This paper proposes an inversion of that approach, starting instead with a deep exploration of local strengths, practices, and knowledge systems. The premise that “the answer lies within” suggests that effective, contextually relevant solutions may already exist within communities. Crucially, this approach requires inclusive engagement with diverse community knowledge holders. This is the essence of positive deviance (PD).

Globally, PD has demonstrated significant impact across a range of health domains. In Vietnam, the PD/Hearth program successfully rehabilitated malnourished children by leveraging locally available foods and caregiver practices, resulting in sustainable, community-led nutrition improvements [9,10,11]. In Upper Egypt, the I MPRESS initiative reduced low-birthweight rates by promoting locally practiced maternal health behaviours [12]. Similarly, Save the Children’s PD cycles in Haripur District, Pakistan, improved maternal and neonatal outcomes by identifying and amplifying effective caregiving practices already present in the community [13].

Within the F1D context, PD has been applied to key areas such as maternal nutrition, early and exclusive breastfeeding, safe and diverse complementary feeding, responsive caregiving, and hygiene practices. These examples underscore PD’s potential to uncover and scale locally grounded strategies that promote child health and development, particularly in settings where conventional interventions have struggled to achieve lasting impact. It is not uncommon for new mothers to feel overwhelmed, disempowered, and isolated during the perinatal period. However, the PD approach offers a powerful reframing of their narratives. By uncovering successful and culturally resonant practices that already exist within their own communities, mothers can rediscover a sense of agency and hope, realizing that they possess the capacity to overcome challenges despite perceived or actual limitations.

The strength of the PD framework lies in its ability to identify and amplify small, high-impact behaviours, whether related to hygiene, nutrition, or caregiving, that yield significant outcomes. This effectiveness is further reinforced by the “it takes one to know one” principle: the nuances of motherhood are often best understood and modelled by those who have lived the same experience. Rooted in the belief that solutions already exist within the community, PD becomes especially powerful during the perinatal period, a time when families undergo profound transitions and rely heavily on informal networks for support. Identifying these local “positive deviants” not only empowers mothers but also transforms everyday practices into sources of collective strength and resilience.

## 3. Leveraging Positive Deviance for a Thriving Community

At its core, PD is a community-driven approach that identifies, understands, and disseminates successful strategies already practiced by individuals who thrive despite facing similar challenges as their peers. When applied consistently to support young children during the F1D, PD has the potential to be one of the most transformative contributions a community can make toward becoming truly child-centred, ensuring that every child can flourish. The effectiveness of PD is readily understood when viewed against the backdrop of well-established theories of human behaviour. For example, people’s tendency to learn from others aligns with Social Learning Theory [14]; the influence of contextual environments on individual and collective behaviour reflects Ecological Systems Theory [15]; and the human drive toward agency and self-determination resonates with Empowerment Theory [16]. Together, these frameworks illuminate how PD fosters behaviour change; individuals observe others, learn from their successes, assume greater control over their actions, and, through environmental alignment, drive positive transformation. In this sense, PD can be seen as a synthesis of established social theories, which contributes to its conceptual depth and enduring appeal.

In public health, PD refers to individuals or groups whose behaviours or outcomes stand out positively despite adverse conditions. These “outliers” succeed not because of external advantages, but due to uncommon yet effective practices that are often overlooked. Crucially, PD recognizes that communities possess latent knowledge and expertise that, when surfaced and shared, can catalyse broader improvements in wellbeing.

The central challenge lies in capturing these “pearls of wisdom”, the locally effective behaviours that contribute to optimal child development and making them accessible to all. A variety of participatory methods have been proposed to uncover and share these insights, including storytelling, yarning, hearth or kitchen table conversations, and community education sessions [9]. These approaches not only surface valuable practices but also foster trust, inclusion, and cultural relevance.

Historical examples underscore PD’s enduring relevance. In the early 1970s, Wray (1972) documented mothers who successfully nourished their children under extremely challenging socioeconomic conditions [17]. He observed:

*“Such mothers, it would appear, know more than we professionals do. They know how, in that incredible environment, to provide their children with basically adequate diets and to protect them from too frequent infections. Perhaps they can teach us. At the very least, we ought to search out the successful mothers in such circumstances, examine their childcare practices, and try to identify what it is they are doing that makes the difference in their children. If we cannot teach these things to other mothers in that environment, perhaps they can.”*

This insight remains foundational to PD, solutions to common problems often already exist within communities and are practiced by those who succeed where others struggle. Because these behaviours are locally grounded, they are inherently acceptable, feasible, and sustainable making them more likely to be adopted and scaled across similar settings.

To operationalize PD, two key frameworks are frequently employed: the 4Ds framework [18] and the Bradley et al. framework (2009) [19]. The 4Ds (or expanded 6Ds) outline a systematic process:

1 **Define** the problem.
2 **Determine** the existence of positive deviants.
3 **Discover** the uncommon but successful strategies.
4 **Design** interventions to enable others to adopt these behaviours.

These frameworks provide a structured pathway for translating individual success into collective wellbeing, reinforcing the notion that communities are not merely recipients of aid but active agents of change.

## 4. Learnings from the Northwest First 1000 Days (NW F1D) Project

The NW F1D project in North-West Tasmania builds on the earlier CAPITOL project initiatives aimed at improving child health and preventing obesity [20,21]. This parent-led, co-designed initiative emphasizes four interconnected domains or quadrants: connection, nutrition, caring, and moving to enhance parenting support, self-efficacy, and early brain development. Through the activation of community hubs, strengthened cross-sector collaboration, and expanded access to evidence-based resources, the NW F1D project aligns with the Healthy Tasmania Strategic Plan and exemplifies a strengths-based, sustainable approach to early childhood wellbeing [22].

The project focused on three local government areas (LGAs) in northwest Tasmania, Burnie, Devonport, and Circular Head, each facing persistent structural and socioeconomic challenges that adversely affect health outcomes. Burnie and Devonport, as regional centres, offer moderate service access but contend with socioeconomic disadvantage and the legacy of industrial decline. Circular Head, a remote rural area centred on Smithton, experiences geographic isolation and limited availability of services. These pressures, compounded by a dispersed population and underdeveloped preventive health infrastructure, contribute to heightened vulnerability to lifestyle-related diseases and perpetuate a deficit-based health narrative.

Central to the NW F1D project was the identification of local success stories—essentially, examples of PD—, though not explicitly labelled as such, given that PD was approached more through a retrospective, interpretive lens. Community storytelling emerged as a key mechanism for surfacing locally grounded examples of successful engagement. These included strong cross-sector collaboration, empowered families sharing knowledge, and high participation in early intervention programs such as Tactical Tots, Mother Goose, and Bubs on Board. Parent education initiatives like Baby and Me and 9 Magic Minutes, alongside nutrition modelling and accessible services, including transport, drop-in allied health clinics, and helplines, demonstrated how community-driven strategies can yield meaningful outcomes for families.

To clarify the storytelling process, methods of identifying positive deviants, mutual learning dynamics, ethical safeguards, and community dissemination, the following summary outlines the key features of the F1D storytelling phase. Positive deviance examples were identified through peer nomination and practitioner observation during the storytelling phase, with all participation entirely voluntary. Parents and practitioners engaged in a genuine exchange of insights, as all involved learned context-specific strategies from the lived experiences of families while facilitating discussions designed to surface constructive, community-led knowledge in a spirit of mutual learning. These deeply collaborative discussions were grounded in dignity, inclusivity, and autonomy, with the workshops fostering safe, empowering spaces where parents, many from disadvantaged backgrounds, could share and co-create personal narratives.

Across the three sentinel sites, the process created welcoming, culturally sensitive environments that encouraged participants to share, interpret, and debate information in relatable yet respectful ways. Storytelling followed three reflective stages: *storymaking* (shaping lived experiences), *listening and holding* (cultivating empathy and understanding), and *storypolishing* (refining narratives with expert insight for community sharing). Stories were always shared voluntarily, with care for participants’ emotional well-being and ownership of their narratives. More than fourteen workshops were held, with parents participating with remarkable openness and commitment. In **Burnie**, the Child and Family Learning Centre facilitated daytime workshops for mothers and evening sessions for fathers, using creative materials and the first 1000 days quadrant framework to prompt reflection on what supports, challenges, and relationships shaped their parenting journeys. Where group settings were unsuitable, family support workers held one-on-one conversations guided by the same quadrant-based questions. Staff observed that the framework provided a gentle entry point for meaningful dialogue around parenting and early development. In **Devonport**, informal sessions led by Community House and Child and Family Learning Centre staff created relaxed spaces for parents to share their stories while children played nearby. Using open-ended prompts, the project ‘Co-Design and Implementation Lead’ encouraged rich, candid storytelling. In **Circular Head**, local partnerships enabled one-to-one interviews at community venues such as Giggles Childcare and the town library, while the Circular Head Aboriginal Corporation broadened participation through an anonymous online ‘Fast Five’ survey. This simple, confidential format captured powerful, emotionally resonant insights, highlighting the value of flexible, locally led approaches to gathering parent stories. Importantly, many described profound personal growth (increased self-awareness, empowerment, and confidence in their parenting, etc.) while the safe, non-judgmental atmosphere reduced shame and encouraged honest dialogue. These insights translated into tangible change, as families adopted healthier routines and more mindful parenting practices.

Embodying a “by the people, for the people” approach, participants shared and learned from one another, demonstrating participatory design in its truest form. Their stories lived beyond the workshops through community storytelling artefacts (e.g., artworks and storyboards displayed in local spaces) that inspired other organizations and university students to embed F1D principles in their own programs. In doing so, parents became informal advocates and leaders, amplifying awareness of early childhood health and development across their communities.

One example of positive deviance emerged from the *Families in Devonport (F1D)* storytelling initiative. A parent participant described how the project transformed engagement from tokenistic consultation to genuine co-creation:

*“I got involved in F1D because I wanted to be part of something real. Something that gives parents a voice and creates space for stories that matter. Whether it’s been through workshops, events, or just honest conversations, I saw that this project wasn’t about ticking boxes it was about connection, change, and community. I’ve stayed involved because the work matters. It’s powerful. It’s helping to shift the culture in Devonport especially for families, young people, and those often overlooked. We’re seeing people find their voices, build confidence, and connect in ways that strengthen the entire community. The most positive part of the process has been the sense of unity and empowerment. F1D has brought together parents from all walks of life to listen, learn, and lead. As a parent, I’ve felt heard, and I’ve seen others step into spaces they never thought they belonged in. There’s been healing, growth, and collaboration. It’s built trust not only between individuals, but also between community members and the systems that are meant to support us.”*

This story illustrates how shifting power to parents, inviting them to lead, not just participate, fostered trust and cultural change within the community. The deviant behavior here was the **co-production of local initiatives by families themselves**, which disrupted established service hierarchies. Over time, these practices spread through schools and services, supported by ongoing storytelling and partnership forums. As another participant reflected:

*“There have definitely been challenges. For many of us, sharing our stories or speaking out can be confronting, especially when there’s been a history of not being heard. There’s also the reality of burnout many community members are giving so much of themselves, often without formal support or resources. And at times, it’s been frustrating knowing the momentum could stall without long term backing. We can’t afford to lose what we’ve started. I’d love to see F1D become a permanent part of Devonport not just as a project, but as a mindset across schools & services. We need ongoing opportunities for community-led storytelling, creative projects, and safe spaces for families and young people. To ensure the messages and momentum continue, we need sustained investment in people, in training, in local leadership. That means funding, yes, but also partnership with government, councils, and services that listen and wish to co-create alongside us. This isn’t just a project; it’s a movement. And for the sake of our children and future generations, we must keep it alive. F1D has already planted seeds, now we need help to keep watering them”*

These reflections highlight the key elements of positive deviance within the project: parents leading rather than being led; relationships built on trust and co-production; and the emergence of a culture of empowerment that spread across schools and services. Although detailed outcomes are discussed in a companion paper, the impacts reported by participants—greater confidence, connection, and collective ownership—demonstrate how such deviance translated into community-level change.

While the formal PD framework was not overtly applied, its principles were embedded within the operational dynamics of the project. The initial “problem definition” phase functioned as a participatory needs analysis, allowing communities to articulate priorities and concerns. Stakeholders emphasized the need for foundational knowledge on F1D principles such as brain development and toxic stress, delivered in accessible formats that were not overwhelming. They also highlighted the importance of addressing parental mental health and acknowledged disparities in access to traditional information networks, particularly among Aboriginal, Torres Strait Islander, and culturally and linguistically diverse (CALD) families. Importantly, it should be noted that all instances of collaboration within the project entailed representation from Indigenous and CALD groups or their proxies. For instance, many of the workshops were attended by stakeholders from the Circular Head Aboriginal Corporation, Indigenous Program Leads from Department for Education, Children and Young People, and on occasion staff affiliated with the Migrant Resource Centre.

Structural challenges, including financial strain and limited support for high-risk pregnancies, were also identified. In response, stakeholders advocated for multi-channel information dissemination through trusted professionals and community leaders, as well as strategies to ensure sustainability, institutional integration, and continuous feedback loops to support long-term program success.

Importantly, the identification of **“positive deviants”** was a central feature of stakeholder engagement. Their stories, surfaced through a dedicated storytelling phase, provided practical examples for communities to learn from and build upon. Once needs were defined, resources mapped, and successful behaviours identified, a co-design approach was employed to develop contextually relevant solutions, aligning with the “discover” and “design” stages of the PD framework.

## 5. Conclusions

Harnessing PD as a guiding principle to support young families during the F1D offers a compelling opportunity to catalyse meaningful, community-led change. By identifying and amplifying local success stories, fostering participatory engagement, and strengthening self-efficacy, communities can implement sustainable, culturally relevant solutions that give every child the best possible start in life. Moving forward, embracing community-driven approaches will be essential to building child-inclusive societies that fully leverage local expertise and strengths.

However, it is critical to remain realistic about the scope and limitations of PD. While the approach rightly celebrates the successful actions of individuals and families, there is a risk of overstating personal agency and underestimating the impact of structural barriers such as poverty, systemic inequities, and limited access to services that constrain many. Findings from the NW F1D project affirm these challenges, underscoring the importance of avoiding narratives that inadvertently blame families who “can’t do more.”

Identifying genuine positive deviants requires careful discernment. Chance or context-specific advantages can be mistaken for replicable behaviours, highlighting the need for rigorous, consultative engagement with communities. Currently, PD identification varies widely, ranging from objective health outcomes (e.g., growth metrics, infection rates) to self-reported behaviours (e.g., breastfeeding duration) and reputation-based nominations. To strengthen methodological rigor, greater standardization is needed including clear criteria for defining PD, appropriate use of controls, transparent documentation of community engagement, and reliance on validated tools and comparison groups where possible [23].

Context and culture remain central. Practices that succeed in one setting may not translate to another, making inclusive representation, particularly of Indigenous and CALD communities, essential to ensuring relevance and feasibility.

The ethics of storytelling also demand careful attention. Highlighting exemplary cases requires informed consent, protection of privacy, and sensitivity to avoid tokenism or romanticization. Success stories should be viewed as dynamic entities, capable of evolving and generating new insights over time.

A pragmatic lens on PD is recommended. This includes evaluating not only ultimate health outcomes but also intermediate indicators such as behaviour adoption, knowledge shifts, and enabling environmental or structural conditions. Such an approach allows for more nuanced understanding of where interventions succeed or fall short.

In sum, PD holds broad applicability in health research and practice, offering valuable insights into “what works” in real-world, resource-constrained settings. For the methodology to mature and its findings to become more reliable and transferable, continued refinement is needed, particularly in conceptual clarity, methodological rigor, and transparency in community involvement.

## Data Availability

This manuscript does not constitute a data paper. Details of the NW F1D study are available from the authors upon reasonable request.

## Abbreviations

The following abbreviations are used in this manuscript:

ABCD	Asset Based Community Development
CBPR	Community Based Participatory Research
CAPITOL	Critical Age Periods Impacting the Trajectory of Obesogenic Lifestyles
F1D	First 1000 days
NW F1D	Northwest First 1000 Days
PD	Positive deviance
CALD	Culturally and linguistically diverse
